# Proposal for a Structured Outpatient Clinic for Dupilumab Treatment in Chronic Rhinosinusitis with Nasal Polyps in the First Year of Treatment

**DOI:** 10.3390/jpm12101734

**Published:** 2022-10-19

**Authors:** Sara Torretta, Eugenio De Corso, Nicolò Nava, Francesca Fraccaroli, Silvia Mariel Ferrucci, Stefano Settimi, Claudio Montuori, Davide Paolo Porru, Camilla Spanu, Giuseppe D’Agostino, Angelo Valerio Marzano, Lorenzo Pignataro

**Affiliations:** 1 Fondazione IRCCS Ca’ Granda Ospedale Maggiore Policlinico, Center of Excellence of Type 2 Inflammation, 20122 Milan, Italy; 2 Department of Clinical Sciences and Community Health, Università degli Studi di Milano, 20122 Milan, Italy; 3 Unit of Otorhinolaryngology-Head and Neck Surgery, “A. Gemelli” Hospital Foundation IRCCS, 00168 Rome, Italy; 4 Faculty of medicine, Università degli Studi di Milano, 20122 Milan, Italy; 5 Department of Head and Neck and Sensory Organs, Catholic University of the Sacred Hearth, 00168 Rome, Italy; 6 Department of Pathophysiology and Transplantation, Università degli Studi di Milano, 20122 Milan, Italy

**Keywords:** chronic rhinosinusitis with nasal polyps, dupilumab, otolaryngology, clinical diagnostic flowchart

## Abstract

Chronic rhinosinusitis with nasal polyps (CRSwNP) is a common disease of the nose and paranasal sinuses with important economic and sanitary burdens, as well as having a great impact on patients’ quality of life. In this field, a new therapeutic approach for those patients who have been described as affected by severe uncontrolled CRSwNP, resistant to medical and best surgical treatment, is represented by subcutaneous human monoclonal antibodies (including dupilumab) that block specific targets involved in the type 2 inflammatory pathway which most commonly drives CRSwNP pathophysiology. This paper aims to report our experience in the management of severe uncontrolled CRSwNP and, in particular, describe our diagnostic workup including baseline evaluation and follow-up visits in the first year of treatment. We also describe into detail our multidisciplinary approach to the disease. We finally report the outcomes of treatment in a real-life setting. In this outpatient real-life setting, our results confirmed the effectiveness of dupilumab in reducing the volume of nasal polyps and restoring nasal obstruction and sense of smell, as well as improving patients’ quality of life. The adherence to the dupilumab treatment was very high. The dose of administration was never modified in patients in the first year of treatment. All the patients respected the plan of the visits at proposed time points. We believe that the structural organization of our outpatient clinic appears to be functional: it allows us to study patients thoroughly before starting treatment and to make a proper follow-up after it starts. We believe that sharing both our strict clinical flowchart and growing experience with dupilumab with the medical community can lead to more standardized and effective pathways of care for CRSwNP patients.

## 1. Introduction

Chronic rhinosinusitis with nasal polyps (CRSwNP) is a common disease with important economic and sanitary burdens, as well as having a great impact on patients’ quality of life given the presence of unpleasant chronic symptoms such as nasal obstruction, loss of smell, rhinorrhea, and sinus pain [1]. CRSwNP is mainly driven by intrinsic mucosal inflammation and recurrence is very common, despite continuous medical treatment and surgical management, especially in cases driven by a type 2 inflammatory pathway [2,3,4]. Inflammation following a mucosal barrier can be divided into type 1 (which targets viruses), type 2 (which targets parasites), and type 3 (which targets extracellular bacteria and fungi). Following EPOS 2020 indications, CRS can be divided into type 2 and non-type 2, where the former is characterized by the presence of cytokines IL-4, IL-13, and IL-5 and the recruitment of eosinophils and mast cells. Type 2 inflammation in CRS has been found to be more severe and resistant to therapies and surgery [1].

Until very recently, the mainstay of therapy for CRSwNP was only limited to corticosteroids (local and systemic) and endoscopic sinus surgery. For those cases that have been defined as severe uncontrolled CRSwNP, a new therapeutic approach is constituted by subcutaneous human monoclonal antibodies, which act on specific cytokines to block type 2 inflammation [2]. In particular, the use of dupilumab has been shown to bring important benefits to patients affected by CRSwNP, both in randomized clinical trials [5] and real-life studies [6,7]. These studies confirmed that dupilumab is effective in improving objective and subjective outcomes, by reducing symptoms and improving both the clinical and radiological framework [2,3,4,5]. Given these data, the Italian Agency of Medicines (AIFA–Agenzia Italiana del Farmaco) recently (November 2020) approved the use of dupilumab in patients with severe uncontrolled CRSwNP, documented by a SinoNasal Outcome Test (SNOT-22) score ≥ 50 and/or Nasal Polyp Score (NPS) ≥ 5, resistant to at least two cycles of systemic corticosteroid therapy in the past 12 months and/or a history of endoscopic sinonasal surgery (ESS) [8].

It is important to note that patients with CRSwNP often have comorbidities such as asthma, atopic dermatitis, eosinophilic otitis, and eosinophilic esophagitis. In particular, recent studies have shown how type 2 (T2) inflammation can be the common underlying cause of these pathologies [1,2]. Dupilumab, which binds the alpha subunit of IL-4 receptors (IL-4Rα type 1 and type 2), thus inhibiting the signaling of IL-4 and IL-13 acting on the IL-4 and IL-13 receptors, can block type 2 inflammation, leading to a significant improvement of both nasal polyps and other T2-related comorbidities. Based on this, dupilumab is largely used in several T2-related diseases, including asthma and atopic dermatitis. In this context, the necessity of taking charge of this category of patients is clear, allowing us to thoroughly study the problems of each patient and to present the most effective pathway of care [9,10]. Based on these considerations, in our hospitals, we created an otolaryngological outpatient clinic dedicated to CRSwNP patients (with or without associated T2-comorbidities) who were candidates for dupilumab treatment and connected to a multi-specialist clinical network, aiming at globally evaluating all patients with any type 2 disorder. This group shared the scientific and clinical efforts focusing on improving the standard of care in patients with type 2 comorbidities, especially those who are candidates for biologic treatment.

This paper aims to describe our experience in the management of severe uncontrolled CRSwNP during the first year of treatment. In particular, we aimed to describe our diagnostic workup, in every step from the baseline to the most important follow-up visit (6 and 12 months). We therefore reported the outcomes of treatment in a real-life setting.

## 2. Materials and Methods

This is a non-profit, real-life observational study. We retrospectively evaluated the charts of 80 prospectically enrolled patients (33 females, 47 males, mean age: 51.58, age range: 23–78) with severe uncontrolled CRSwNP, as defined by EUFOREA [11], who were followed and treated at our institutions (the Center of Excellence of Type 2 Inflammation of Fondazione IRCCS Ca’ Granda Ospedale Maggiore Policlinico, Milan, Italy, and “A.Gemelli” Hospital Foundation IRCCS, Rome, Italy) with dupilumab 300 mg administered subcutaneously every 2 weeks as add-on therapy to intra-nasal corticosteroids. The severity of disease was graded on the basis of subjective symptoms (based on SNOT-22), endoscopic appearance (NPS), and the failure of previous systemic corticosteroids (two cycles in the past 12 months) and/or ESS.

Exclusion criteria were: patients refusing to undergo treatment with biologics, patients with known immune defects and/or autoimmune diseases, and patients refusing to undergo complete follow-up. The patients were followed starting in February 2021. The study was conducted in accordance with the Declaration of Helsinki and approved by the Ethics Committee of “Milano Area 2” (protocol no. 0003629).

**Figure 1:**
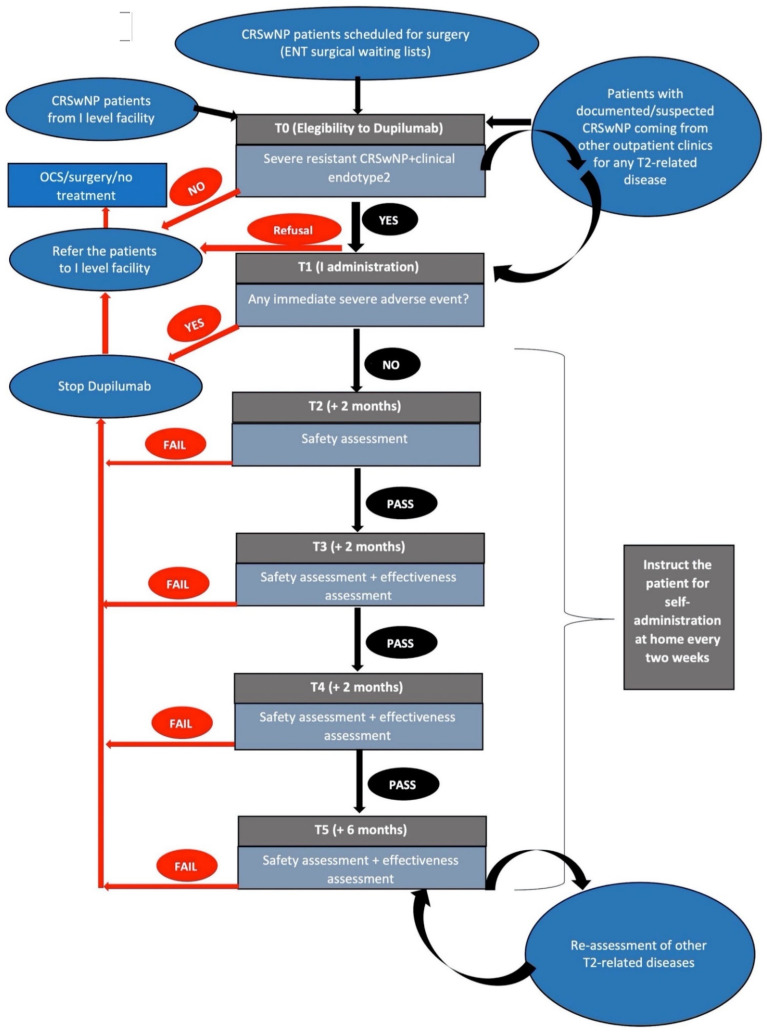
Flow-chart of patients within the outpatient clinic. Abbreviations: CRSwNP: chronic rhinosinusitis with nasal polyps; ENT: ear, nose, and throat; OCS: oral corticosteroids. Legend: T0 (baseline evaluation), T1 (first administration), T2 (follow-up evaluation after 2 months of treatment), T3 (follow-up evaluation after 4 months of treatment), T4 (follow-up evaluation after 6 months of treatment), T5 (follow-up evaluation after 12 months of treatment).

Patients to be screened for dupilumab treatment were selected from our surgical waiting lists or from our first-level Ear nose and throat (ENT) outpatient facility or any other outpatient clinics within our hospital for any T2-related disease (i.e., pneumological, allergological, dermatological, gastroenterological facility) (Figure 1).

After collecting the patient’s case history, the first ENT examination (Time 0 or T0) focuses on confirming the presence of a severe and resistant CRSwNP and defining the endotype in each patient based on international guidelines [1,2,3,4,5,6,7,8,9,10,11].

In order to do so, each patient undergoes complete ENT assessment with rigid HD nasal video-endoscopy (with definition of the NPS [1,2,3,4,5]), SNOT-22 questionnaire [1,2], and sense-of-smell assessment [1,2,3,4,5]. Previous control of the disease by surgery or systemic corticosteroids was also assessed to evaluate eligibility for dupilumab treatment [12,13]. Computed-tomography (CT) scans (performed no more than 3 months earlier) are evaluated and the Lund–Mackay score (LMS) is defined [1].

On T0, the patients also perform wide-screening blood tests, a complete urine test, and nasal cytology. The first wide blood test includes a complete blood count with formula, lymphocyte subpopulation analysis, eosinophils count; complete liver enzymes; serum protein; serum creatinine; serum reactive C-protein (CRP); serum IgA, IgG, IgM, IgE level; serum peri-nuclear anti-neutrophil cytoplasmatic antibodies, extractable nuclear antigens; creatine phosphokinase serum level, eosinophil cationic protein serum level (ECP). The results of these tests allow us to study the underlying clinical endotype and to make a differential diagnosis with other immunological and autoimmune diseases, as well as primary immunodeficiencies and IgG4-related diseases.

We suggest that prescribing specialists’ examinations actively search for comorbidities such as asthma, atopic dermatitis, and eosinophilic esophagitis (Figure 1). Finally, we usually recommend ophthalmologic evaluation with a study of the whole ocular surface at the beginning of treatment, with conjunctivitis being one of the most common side effects of dupilumab (especially in patients with atopic dermatitis [14]).

Dupilumab treatment was prescribed following AIFA’s treatment plan indications, which are: age ≥ 18 years old; severe CRSwNP defined by: SNOT-22 ≥ 50 and/or NPS ≥ 5; uncontrolled disease defined as failure of previous systemic corticosteroids (two cycles in the past 12 months) and/or previous ESS.

We usually preform first administration on T1 in the presence of healthcare personnel in our outpatient department, to monitor any possible immediate adverse effects (e.g., allergic reactions). Moreover, it is important to educate patients for the following self-administrations, to be performed by patients themselves at home.

Clinical evaluations include complete ENT assessment with rigid HD nasal video-endoscopy (and NPS definition), SNOT-22 questionnaire, 0 to 10 Visual Analogue Scale (VAS) referred to the main sinonasal symptoms, Nasal Congestion Score (NCS), sense-of-smell assessment, nasal cytology, recording the possible impact of dupilumab on comorbidities, and restricted blood control tests.

**Figure 2:**
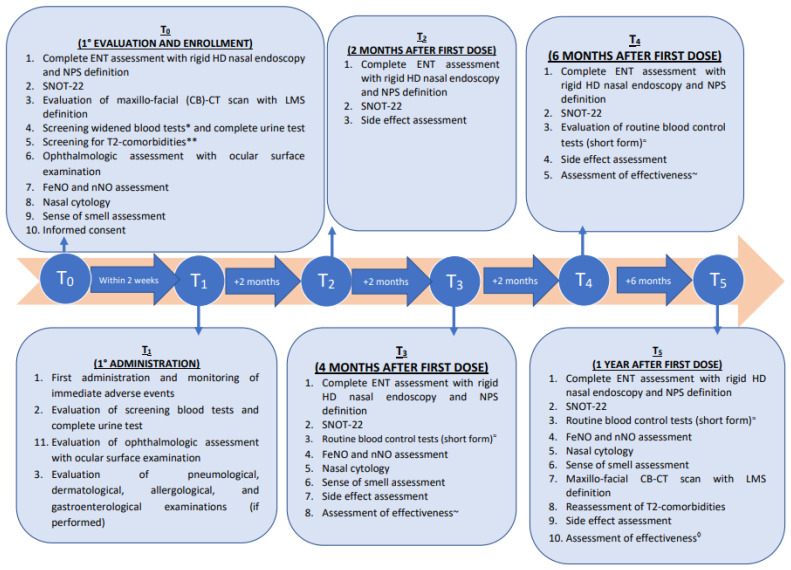
Flow-chart describing the diagnostic and therapeutic program scheduled for patients with severe chronic rhinosinusitis with nasal polyps (CRSwNP) and who are candidates for dupilumab. *Abbreviations: ENT: ear, nose, and throat; NPS: Nasal Polyp Score; LMS: Lund–Mackay score; SNOT-22: SinoNasal Outcome Test score; CB-CT: cone-beam computed tomography; FeNO: fraction of exhaled nitric oxide; nNO: nasal nitric oxide.* *: including complete blood count and differential, lymphocyte subpopulation analysis, eosinophils count; complete liver enzymes; serum protein; serum creatinine; serum reactive C-protein; serum IgA, IgG, IgM, IgE level; serum peri-nuclear antineutrophil cytoplasmatic antibodies, extractable nuclear antigens; creatine phosphokinase serum level, eosinophil cationic protein serum level; **: in case of suspected or known T2-related comorbidities address the patients to further (dermatological, allergological, pneumological, and gastroenterological) assessments; ~: based on documented improvement in NPS and/or SNOT-22 and/or sense of smell assessment; ≈: including complete blood count and differential, eosinophils count; serum IgE level; eosinophil cationic protein serum level; ◊: based on documented improvement in NPS and/or SNOT-22 and/or sense of smell assessment and/or LMS and/or comorbidities.

Our protocols of follow-up visits, including the parameters we evaluate at each timepoint to assess the effectiveness and tolerability of treatment, are described in Figure 2.

## 3. Results

### Effectiveness of Dupilumab on Reducing Polyps’ Volume and Restoring Nasal Congestion

**Figure 3:**
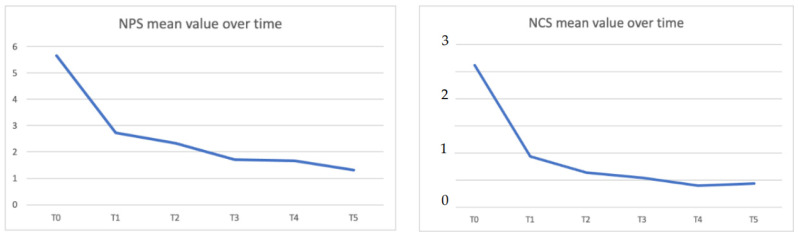
NPS (nasal polyps score) and NCS (nasal congestion score) mean values over time.

Dupilumab has been shown to be effective in reducing polyps’ volume (measured with Nasal Polyp Score) and restoring nasal congestion (measured with Nasal Congestion Score). The mean NPS score decreased significantly from 5.66 ± 1.42 at baseline to 1.71 ± 1.82 at 6 months (*p* < 0.05) and to 1.31 ± 1.70 at 12 months (*p* < 0.05). The mean NCS score decreased significantly from 2.63 ± 0.64 at baseline to 0.54 ± 0.64 at 6 months (*p* < 0.05) and 0.44 ± 0.63 at 12 months (*p* < 0.05). Figure 3 shows the NPS and NCS mean values over time.

Furthermore, we observed a significant improvement in quality of life measured by several indicators. We observed an average reduction in SNOT-22 from 60.11 ± 18.66 at baseline to 18.61 ± 15.76 at 6 months of treatment and to 13.49 ± 10.87 at 12 months (*p* < 0.05).

Total VAS (Visual Analog Scale) mean value decreased from 6.82 ± 1.77 at baseline to 1.67 ± 1.52 at 6 months (*p* < 0.05) and 1.33 ± 1.12 at 12 months (*p* < 0.05).

**Figure 4:**
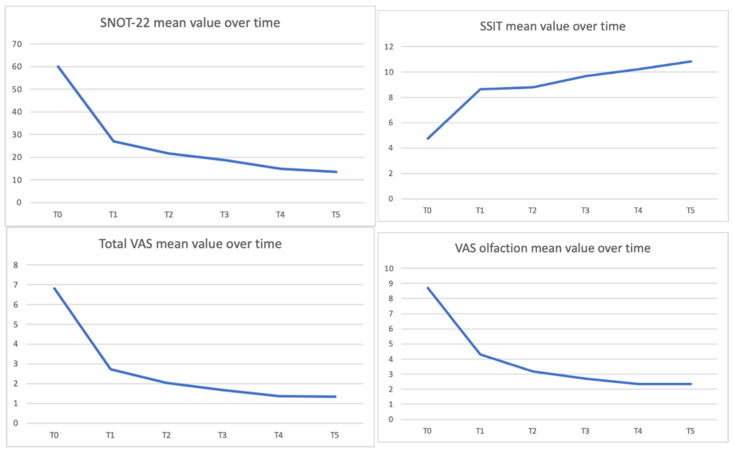
SNOT-22 (sinonasal outcome test),SSIT (sniffing sticks identification test), Total VAS (visual analogue scale) and VAS olfaction mean values over time.

We also observed an improvement of olfaction measured with Sniffin’ Sticks-16 Identification Test (SSIT-16) and VAS olfaction: the SSIT-16 mean score improved from 4.73 ± 3.95 at the baseline to 9.69 ± 3.55 at 6 months of treatment. This positive trend was confirmed by the analysis at 12 months, with an increase in olfactory performance to 10.85 ± 3.81 for mean SSIT-16 (*p* < 0.05). We also observed that mean VAS olfaction values decreased from 8.7 ± 1.93 at baseline to 2.71 ± 3.11 at 6 months (*p* < 0.05) and to 2.36 ± 3.11 at 12 months (*p* < 0.05). Figure 4 shows SNOT-22, SSIT, Total VAS, and VAS olfaction mean values over time.

The adherence to treatment with dupilumab was very high. The dose of administration was never modified for patients in the first year of treatment. All the patients respected the plan of the visits at proposed time points.

Finally, we evaluated patients’ clinical response according to EPOS guidelines. At 6 months of follow-up, 2/80 patients (2.5%) showed no clinical response, 2/80 patients showed a “poor” response, 34/80 (42.5%) had a “moderate” response, and 42/80 (52.5%) had an “excellent” response. At 12 months of treatment, 4 patients (5%) had no clinical response, 2 patients showed a “poor response”, 19/80 patients (23.75%) showed a “moderate” response, and 55/80 patients (68.75%) had an “excellent” response. All 4 patients who had no clinical response stopped treatment with dupilumab at 12 months of follow-up; in addition, 1 patient was lost to follow-up and 2 patients underwent salvage surgery for nasal polyposis.

In relation to safety concerns, dupilumab was well tolerated by all patients in the study. No severe adverse reactions were reported: 7 patients reported the onset of hypereosinophilia (>1500 cells/microL); 2 patients reported the onset of arthralgia associated with hypereosinophilia and had to stop treatment; 1 patient reported the onset of migraine that positively resolved within 24 h without medication; and 3 patients reported minor symptoms such as conjunctivitis, which occurred within the first month of treatment, with spontaneous resolution and without the need for medical treatment.

## 4. Discussion

In this manuscript, we described our experience in the management of severe uncontrolled CRSwNP in our outpatient clinic setting. For what concerns our diagnostic workup, we believe that baseline evaluation is essential to define phenotype and endotype, selecting patients with uncontrolled disease. At the baseline, it is very important to screen patients for the presence of any possible concomitant T2-related disease, addressing, if necessary, the patients’ further evaluation (dermatological, allergological, pneumological, and gastroenterological) aimed at placing them at the center of an integrated and comprehensive multi-specialist clinical network to cure them from a 360-degree point of view.

Our proposal of follow-up during the first year of therapy consists of bimestrial ORL examinations for the first 6 months and then at 1 year after the beginning of therapy (Figure 1). Effectiveness and safety are evaluated during each follow-up visit.

We think that the strict clinical follow-up of the first months of treatment is effective in monitoring adverse events or any other problem that patients may have (i.e., difficulties in the administration, common and mild adverse events that can create anxiety in patients, simultaneous administration of other drugs or vaccines).

On T3 and T4, the effectiveness assessment will consider a documented improvement in NPS and/or SNOT-22 and/or sense-of-smell assessment; otherwise, on T5 we consider a possible improvement in any other T2-related comorbidity and we suggest, in selected cases, the LMS obtained at a control maxillo-facial cone-beam computed tomography (CB-CT) scan. For better monitoring (especially the development of eosinophilia as a possible side effect), we suggest a restricted panel of blood tests 4 (T3) and 8 (+2 months from T4) months after the beginning of treatment. This panel includes complete blood count with formula, ECP, and total IgE. On T3, we will also repeat the nasal cytology sampling.

In the case of development of any severe adverse event or inadequate effectiveness as defined by international guidelines^1,4^, dupilumab treatment is discontinued and the patient is referred to our first-level ENT facility.

Our data confirm the effectiveness of dupilumab in a real-life setting: as already stated in our previous experience [6,7,8,9,10,11,12,13,14,15], the results in real life seem to be better than the results in the trials.

Here, we indeed observed an improvement in all considered variables; more specifically, dupilumab was effective in reducing NPS and restoring nasal obstruction (measured with Nasal Congestion Score), as well as improving patients’ sense of smell (as noted with the improvement in Sniffin’ Sticks Identification Test and VAS olfaction) and quality of life (measured with SNOT-22 and Total VAS). Our data are consistent with those obtained from our previous real-life experience [6], showing even more favorable results in almost all variables considered.

In addition, regarding the evaluation of clinical response according to EPOS guidelines, we noticed how patients were divided in the four groups of clinical response in a similar way to that of a previous study [6].

The limitations of the study line are its retrospective nature and the small sample size; the strengths are the homogeneity of the interventions and the real-life setting.

We believe that the structural organization of our outpatient clinic appears to be functional: it allows us to study patients thoroughly before starting treatment and to make a proper follow-up after starting it. A multidisciplinary network clearly facilitates the classification and the 360-degree treatment of these patients, who often present type 2 comorbidities.

## 5. Conclusions

In conclusion, even though dupilumab has proven to be effective in treating refractory CRSwNP [2,5], no unique protocol of screening and follow-up has yet been created. We believe that our plans and organization for the start and follow-up of treatment with a new emerging therapy such as dupilumab could standardize and make more effective the pathways of care for these patients, who have always been difficult to treat effectively. We believe that sharing our plans and growing experience of dupilumab with the medical community can lead to more standardized and effective pathways of care for CRSwNP patients.

## Data Availability

Data available on request from the authors.

## References

[1] Fokkens W.J., Lund V.J., Hopkins C., Hellings P.W., Kern R., Reitsma S., Toppila-Salmi S., Bernal-Sprekelsen M., Mullol J., Alobid I. (2020). European Position Paper on Rhinosinusitis and Nasal Polyps 2020. Rhinology.

[2] Bachert C., Hellings P.W., Mullol J., Naclerio R.M., Chao J., Amin N., Grabher A., Swanson B.N., Hamilton J.D., Guillonneau S. (2019). Dupilumab improves patient-reported outcomes in patients with chronic rhinosinusitis with nasal polyps and comorbid asthma. J. Allergy Cli. Immunol. Pract..

[3] De Corso E., Baroni S., Settimi S., Onori M.E., Mastrapasqua R.F., Troiani E., Moretti G., Lucchetti D., Corbò M., Montuori C. (2022). Sinonasal Biomarkers Defining Type 2-High and Type 2-Low Inflammation in Chronic Rhinosinusitis with Nasal Polyps. J. Pers. Med..

[4] De Corso E., Baroni S., Onori M.E., Tricarico L., Settimi S., Moretti G., Troiani E., Mastrapasqua R.F., Furno D., Crudo F. Calprotectin in nasal secretion: A new biomarker of non-type 2 inflammation in CRSwNP. Acta Otorhinolaryngol. Ital..

[5] Bachert C., Han J.K., Desrosiers M., Hellings P.W., Amin N., Lee E.S., Mullol J., Greos L.S., Bosso J.V., Laidlaw T.M. (2019). Efficacy and safety of dupilumab in patients with severe chronic rhinosinusitis with nasal polyps (LIBERTY NP SINUS-24 and LIBERTY NP SINUS-52): Results from two multicentre, randomised, double-blind, placebo-controlled, parallel-group phase 3 trials. Lancet.

[6] De Corso E., Settimi S., Montuori C., Corbò M., Passali G.C., Porru D.P., Lo Verde S., Spanu C., Penazzi D., Di Bella G.A. (2022). Effectiveness of Dupilumab in the Treatment of Patients with Severe Uncontrolled CRSwNP: A "Real-Life" Observational Study in the First Year of Treatment. J. Clin. Med..

[7] Cantone E., De Corso E., Ricciardiello F., Di Nola C., Grimaldi G., Allocca V., Motta G. (2022). Olfaction Recovery following Dupilumab Is Independent of Nasal Polyp Reduction in CRSwNP. J. Pers. Med..

[8] De Corso E., Bellocchi G., De Benedetto M., Lombardo N., Macchi A., Malvezzi L., Motta G., Pagella F., Vicini C., Passali D. (2022). Biologics for severe uncontrolled chronic rhinosinusitis with nasal polyps: A change management approach. Consensus of the Joint Committee of Italian Society of Otorhinolaryngology on biologics in rhinology. Acta Otorhinolaryngol. Ital..

[9] Seccia V., D’Amato M., Scioscia G., Bagnasco D., Di Marco F., Fadda G., Menzella F., Pasquini E., Pelaia G., Tremante E. (2022). Management of Patients with Severe Asthma and Chronic Rhinosinusitis with Nasal Polyps: A Multidisciplinary Shared Approach. J. Pers. Med..

[10] De Corso E., Bilò M.B., Matucci A., Seccia V., Braido F., Gelardi M., Heffler E., Latorre M., Malvezzi L., Pelaia G. (2022). Personalized Management of Patients with Chronic Rhinosinusitis with Nasal Polyps in Clinical Practice: A Multidisciplinary Consensus Statement. J. Pers. Med..

[11] Bachert C., Han J.K., Wagenmann M., Hosemann W., Lee S.E., Backer V., Mullol J., Gevaert P., Klimek L., Prokopakis E. (2021). EUFOREA expert board meeting on uncontrolled severe chroni rhinosinusitis with nasal polyps (CRSwNP) and biologics: Definition and management. J. Allergy Clin. Immunol..

[12] De Corso E., Pipolo C., Cantone E., Ottaviano G., Gallo S., Canevari F.R.M., Macchi A., Monti G., Cavaliere C., La Mantia I. (2022). Survey on Use of Local and Systemic Corticosteroids in the Management of Chronic Rhinosinusitis with Nasal Polyps: Identification of Unmet Clinical Needs. J. Pers. Med..

[13] De Corso E., Settimi S., Tricarico L., Mele D.A., Mastrapasqua R.F., Di Cesare T., Salvati A., Trozzi L., De Vita C., Romanello M. (2021). Predictors of Disease Control After Endoscopic Sinus Surgery Plus Long-Term Local Corticosteroids in CRSwNP. Am. J. Rhinol. Allergy.

[14] Akinlade B., Guttman-Yassky E., De Bruin-Weller M., Simpson E., Blauvelt A., Cork M., Prens E., Asbell P., Akpek E., Corren J. (2019). Conjunctivitis in dupilumab clinical trials. Br. J. Dermatol..

[15] De Corso E., Furneri G., Salsi D., Fanelli F., Ronci G., Sala G., Bitonti R., Cuda D. (2022). Cost-Utility Analysis of Dupilumab for the Treatment of Chronic Rhinosinusitis with Nasal Polyps (CRSwNP) in Italy. J. Pers. Med..

